# Mechanical Stretch of High Magnitude Provokes Axonal Injury, Elongation of Paranodal Junctions, and Signaling Alterations in Oligodendrocytes

**DOI:** 10.1007/s12035-018-1372-6

**Published:** 2018-10-08

**Authors:** Elena Chierto, Anne Simon, Francesca Castoldi, Delphine Meffre, Giulia Cristinziano, Francesca Sapone, Alex Carrete, Didier Borderie, François Etienne, François Rannou, Barclay Morrison, Charbel Massaad, Mehrnaz Jafarian-Tehrani

**Affiliations:** 1 INSERM UMR-S 1124, Université Paris Descartes, Sorbonne Paris Cité, Faculté des Sciences Fondamentales et Biomédicales, 45 rue des Saints-Pères, 75006 Paris, France; 2 Service de Diagnostic Biologique Automatisé, Hôpitaux Universitaires Paris Centre - Groupe Hospitalier Cochin (AP-HP), 27 rue du faubourg saint Jacques, 75679 Paris Cedex 14, France; 3 Plateforme de mécanobiologie, Sorbonne Paris Cité, Faculté des Sciences Fondamentales et Biomédicales, Université Paris Descartes, 45 rue des Saints-Pères, 75006 Paris, France; 4 Service de Rééducation et de Réadaptation de l’Appareil Locomoteur et des Pathologies du Rachis, Hôpitaux Universitaires Paris Centre - Groupe Hospitalier Cochin (AP-HP), 27 rue du faubourg saint Jacques, 75679 Paris Cedex 14, France; 5 Department of Biomedical Engineering, Columbia University, 1210 Amsterdam Ave, 351 Engineering Terrace, MC8904, New York, NY 10027 USA

**Keywords:** Tensile strain, Stretch-induced injury, Myelin, Oligodendrocytes, Traumatic brain injury, Cerebellum, Mechanostimulation, White matter injury

## Abstract

Increasing findings suggest that demyelination may play an important role in the pathophysiology of brain injury, but the exact mechanisms underlying such damage are not well known. Mechanical tensile strain of brain tissue occurs during traumatic brain injury. Several studies have investigated the cellular and molecular events following a static tensile strain of physiological magnitude on individual cells such as oligodendrocytes. However, the pathobiological impact of high-magnitude mechanical strain on oligodendrocytes and myelinated fibers remains under investigated. In this study, we reported that an applied mechanical tensile strain of 30% on mouse organotypic culture of cerebellar slices induced axonal injury and elongation of paranodal junctions, two hallmarks of brain trauma. It was also able to activate MAPK-ERK1/2 signaling, a stretch-induced responsive pathway. The same tensile strain applied to mouse oligodendrocytes in primary culture induced a profound damage to cell morphology, partial cell loss, and a decrease of myelin protein expression. The lower tensile strain of 20% also caused cell loss and the remaining oligodendrocytes appeared retracted with decreased myelin protein expression. Finally, high-magnitude tensile strain applied to 158N oligodendroglial cells altered myelin protein expression, dampened MAPK-ERK1/2 and MAPK-p38 signaling, and enhanced the production of reactive oxygen species. The latter was accompanied by increased protein oxidation and an alteration of anti-oxidant defense that was strain magnitude-dependent. In conclusion, mechanical stretch of high magnitude provokes axonal injury with significant alterations in oligodendrocyte biology that could initiate demyelination.

**Electronic supplementary material:**

The online version of this article (10.1007/s12035-018-1372-6) contains supplementary material, which is available to authorized users.

## Introduction

Myelin, the concentrically laminated membrane structure that wraps around axons, is a major component of white matter. It is produced by oligodendrocytes in the central nervous system (CNS). Among its different functions, myelin, essential for rapid impulse conduction, serves as an insulator and as trophic and metabolic support to axons [1]. Increasing clinical and experimental evidences suggest that demyelination may play an important role in the pathophysiology of brain injury [2, 3]. While numerous studies reported the effects of traumatic insult on gray matter and resulting neuronal impairment, additional findings show an equivalent importance of white matter damage post-injury [4–10]. Marked white matter atrophy and degeneration have been observed in patients who survived to a chronic post-injury stage after moderate to severe head injury [5, 9–12]. Other studies in humans and experimental models have also shown widespread myelin loss and oligodendrocyte apoptosis following brain insult [13–18].

In addition, brain injury triggers a complex cascade of metabolic, cellular, and molecular alterations such as increased free radical generation [19]. Excessive production of reactive oxygen species (ROS) results in oxidative stress, which has a significant role in the etiology of progressive neuropathology following brain injury [20]. The CNS is extremely sensitive to free radical insults because of its relatively limited total antioxidant defenses [21, 22]. Among CNS cell types, oligodendrocytes are particularly vulnerable because of their lipid-rich membrane, low glutathione reductase activity, and low level of glutathione (GSH) [23, 24]. The endogenous defense system is able to scavenge and prevent formation of free radicals, protecting tissues from oxidative damage [25]. However, ROS can oxidize macromolecules, such as DNA, proteins, carbohydrates, and lipids under pathological conditions [26]. Therefore, oligodendrocytes are a particularly vulnerable cell type in the context of brain injury.

While the aforementioned reports highlight the vulnerability of oligodendrocytes and myelin damage following brain injury, the pathophysiological mechanisms underlying white matter impairment are not well known. The brain is encapsulated in a rigid skull, and the mechanical response to an impact involves a combination of stretch, compression, and shear. Among the various mechanical forces causing brain damage [27, 28], stretch-induced injury constitutes a major mode of deformation [29]. In fact, it is known that a major force causing injury of nervous tissue is the rapid tissue deformation and strain, or more simply stretch [29]. In addition, a continuous and sustained stress on brain parenchyma could occur following TBI with the formation of cerebral edema, which begins early after the onset of TBI and evolves over hours post-injury. Evolving brain edema leads to the mechanical displacement of brain structures and may cause herniation. Further injury occurs through axonal stretch, vascular disruption or compression, or a combination [30, 31]. While several studies reported the effect of stretch-induced injury on different CNS cell types (neurons, astrocytes, and microglia), our knowledge regarding oligodendrocytes remains limited. It has been demonstrated that oligodendrocyte progenitors and neural stem cells are mechano-sensitive, and mechanical strain of a physiological magnitude promotes cell differentiation [32–39]. However, the effect of mechanical strain of high magnitude that occurs during and after brain trauma on oligodendrocytes remains under investigated. We hypothesized that such strain can initiate oligodendroglial damage and cause demyelination.

In this study, we aimed to decipher the cellular and molecular responses of oligodendrocytes and myelinated fibers to mechanical tensile strain of high magnitude in vitro and ex vivo, respectively. For the latter, we used organotypic culture of cerebellar slices, because the cerebellum is very rich in myelinated fibers; for the former, we used isolated oligodendrocytes from mouse primary culture or the 158N oligodendroglial cell line. We chose a range of tensile strain (20% and 30% engineering strain) relevant to those occurring in humans during rotational acceleration-deceleration injury [40, 41]. In our study, we assessed two hallmarks of brain injury: (i) accumulation of axonal amyloid precursor protein (APP), a marker of axonal injury [42, 43], and (ii) elongation of paranodal junctions which has been shown to occur following traumatic impact [44, 45]. The effect of mechanical tensile strain was also examined on the expression of myelin proteins and on the activation of the mitogen-activated protein kinase (MAPK) signaling pathway, which is known to be particularly stretch-responsive [46–49]. Finally, mechanical tensile strain of different magnitudes was applied to oligodendrocytes to assess cell morphology, cell adhesion, expression of myelin genes and corresponding proteins, activation of MAPK signaling pathway, and redox status.

In this study, we provide evidence that mechanical tensile strain of high magnitude provokes the hallmarks of brain trauma. It causes loss of oligodendrocytes accompanied by alterations of myelin protein expression and MAPK signaling, with an enhancement of ROS production, and deregulation of redox status. Therefore, such mechanical strain initiates axonal injury and molecular alterations that could initiate demyelination and white matter injury.

## Materials and Methods

### Chemical Reagents and Antibodies

Untreated Bioflex® plates (BF-3001U) and laminin Bioflex® plates (BF-3001L) were purchased from Dunn Labortechnik GmbH (Asbach, Allemagne). Poly-L-lysine (P9404), propidium iodide (P4170), Cell Lysis Reagent (C2978), TRITC-phalloidin (P1951), Hoechst (H2261), and anti α-tubulin antibody (T6074) were purchased from Sigma-Aldrich (Lyon, France). Laminin (23017-015), BME (41010-026), DMEM (11960), HBSS (14060-040), Pierce®ECL 2 Western Blotting substrate kit (PI80196), Alexa 488 goat anti-mouse IgG (A21121), anti-APP antibody (51-2700), and CM-H2DCFDA probe (C6827) were purchased from Thermo Fisher Scientific (Courtaboeuf, France). Cy3 goat anti-rabbit (111-165-003) was purchased from Jackson ImmunoResearch (Cambridge, UK). Anti-MAG (MAB1567), anti-MBP (MAB381), anti-phospho-ERK1/2 (05-797R), and anti ERK1/2 (06-182) antibodies were purchased from Millipore (Molsheim, France). Anti-SMI312 (837904) antibody was purchased from Biolegend (San Diego, USA). Anti-Caspr (ab34151) and rabbit polyclonal β-actin (ab8227) antibodies were purchased from Abcam (Cambridge, UK). Anti-PDGFR-α antibody (sc-338) was purchased from Santa Cruz (Dallas, USA). Anti-PLP antibody (NB10074503) was purchased from Novus Biologicals. Anti-JNK (9252), anti-phospho-JNK (9251S), anti-p38 (212), and anti-phospho-p38 (9211) antibodies were purchased from Cell Signaling (Danvers, USA).

### Animals

Organotypic cerebellar cultures and oligodendrocyte primary cell cultures were obtained from 8–10-day postnatal (P8–P10) and P1–P3 wild-type C57Bl/6 mice (Janvier, Le Genest St Isle, France), respectively. Animals were housed in a controlled temperature environment (22 ± 2 °C), under a 12-h light/dark cycle, with access to food and water ad libitum. Animal care and experiments were approved by the Paris Descartes University (CEEA34.MJT.075.12) respecting the French regulations and the European Communities Council Directive of September 2010/63/UE, on the protection of animals used for scientific purposes.

### Organotypic Cerebellar Slice Culture

The culture protocol was used as described previously [50]. The organotypic culture of cerebellar slices on silicone membrane was adapted from a protocol of Morrison and collaborators [51, 52]. Briefly, after decapitation, the cerebellum was dissected out and meninges were removed. Parasagittal slices (350 μm) were cut using a Macllwain Tissue Chopper. Slices taken in the vermis of cerebellum were transferred to Bioflex® Plates pretreated in a UV ozone cleaner (Bioforce Nanosciences, Salt Lake City, USA) and coated with poly-L-lysine (320 μg/mL) and laminin (80 μg/mL). Cultures were maintained in BME and HBSS media supplemented with 25% of horse serum. Every 2–3 days, 50% of culture medium was substituted. Cerebellar slices were cultured at 35 °C in a humidified atmosphere of 5% CO_2_ and maintained on a rocker (2D movement, inclination angle 14°, rocking rate 1 cycle/min) to aid gas exchange and diffusion. To avoid an excessive evaporation of medium, a humidifier was inserted in the incubator and turned on for 3 cycles of 5 min per day, equally distributed within 24 h. Cultures were maintained 7 days in vitro (DIV) before being submitted to a mechanical stretch. Just before stretching, the medium was replaced by fresh medium with 5% horse serum.

### Oligodendrocyte-Enriched Primary Culture

To prepare the oligodendrocyte-enriched primary cultures, oligodendrocytes were collected after 14 DIV from primary mixed glial cell culture obtained from new-born mice (P0–P3), as previously described [53, 54], with slight modifications. Oligodendrocytes growing on the astrocyte layer were detached mechanically, and microglia were eliminated with a pre-plating step. The pellet containing oligodendrocytes was suspended in supplemented Dulbecco’s minimal essential medium (DMEM) with insulin, transferrin, and selenium (BD Biosciences, Franklin Lakes, USA) and 2% heat inactivated fetal bovine serum (HI-FBS). Cells were seeded at 4–5 × 10^5^ cells in Bioflex® plates coated with poly-L-lysine (25 μg/mL). Cells were grown at 37 °C in a humidified atmosphere of 5% CO_2_ for 2 DIV before being submitted to mechanical stretch.

### 158N Oligodendroglial Cell Line Culture

The 158N oligodendroglial cell line, which preserves oligodendrocyte characteristics and expresses myelin proteins [55, 56], was kindly provided by Dr. S. M. Ghandour (Strasbourg, France). Cells were maintained in DMEM supplemented with 5% HI-FBS. Cells were grown at 37 °C in a humidified atmosphere of 5% CO_2_. For the preparation of the cells subjected to mechanical stretch, cells were trypsinized and plated at a density of 3 × 10^5^ cells per well on laminin-coated Bioflex**®** plates. Cells were grown at 37 °C in a humidified atmosphere of 5% CO_2_ for 72 h before being submitted to a mechanical stretch. Twenty four hours before stretching, the medium was replaced with fresh medium containing 1% HI-FBS.

### In Vitro Model of Mechanical Stretch

Cells or cerebellar slices grown on BioFlex® six-well plates were submitted to mechanical stretch using the Flexcell**®** FX 5000™ Tension System (Flexcell International Corporation, NC, USA), a computer-regulated bioreactor that uses vacuum pressure to apply a strain to cells/slices cultured on elastic silicone membranes [57]. Organotypic cerebellar slices were subjected to an equibiaxial static strain of 30% for 30 min. The response was evaluated at time 0 h post-stretch. Cells were exposed to two magnitudes of strain, 30% for 30 min and 20% for 20 h. Control (non-stretched) plates were prepared and underwent the same conditions, except the mechanical strain. All analyses were performed at time 0 h post-stretch. It is noteworthy that the three model systems (cell line, primary culture, slice culture) remained viable at least 24 h after stress induction.

### Flow Cytometric Analysis

158N cells were trypsinized and collected for analysis with a BD FACSCanto™ II flow cytometer. Cells were centrifuged (1500 rpm, 3 min), washed, and suspended at 10^6^/mL of PBS 0.1 M (Ca^2+^ and Mg^2+^ free). For each analysis, 10,000 cells were counted. We considered only the population of cells devoid of aggregates and debris.

In order to evaluate cell viability and ROS production, a double staining with propidium iodide (PI) and carboxy-dichloro-dihydro-fluorescein diacetate (CM-H2DCFDA) was performed [58]. Cells were incubated with 1.25 μM of CM-H2DCFDA probe for 30 min at 37 °C in the dark. Excess probe was washed out and cells were suspended in PBS (Ca^2+^ and Mg^2+^ free) and incubated 10 min with 1 μg/mL of PI at room temperature (RT). DCF fluorescence was recorded in the FL-1 (530/30 nm) channel together with PI fluorescence in FL-2 (585/42 nm). We used N-acetylcysteine (NAC) at 5 mM as a positive control for the antioxidant activity, and *tert*-butyl hydroperoxide (*t*BHP) 100 μM as a positive control for ROS production.

### Biochemical Analysis

158N cells were washed with PBS, trypsinized and centrifuged (600*g*, 10 min). Cell pellets were suspended in 1 mL of Cell Lysis Reagent with protease inhibitor (cOmplete, Mini, EDTA-free, Roche, Bâle, Suisse). Cell lysates were then analyzed for all the following biochemical assays.

#### Determination of Advanced Oxidation Protein Products

Protein oxidation was assessed as described previously [59]. Advanced oxidation protein product (AOPP) concentrations are expressed as micromoles per liter of chloramine-T equivalents.

#### Determination of Carbonyl Levels

Protein carbonyl groups were detected and quantified using 2,4-dinitrophenylhydrazine (DNPH) as previously described [60]. Carbonyl levels were expressed as nanomoles of carbonyl per milligram of proteins.

#### Determination of Superoxide Dismutase Activity

Superoxide dismutase (SOD) activity was evaluated using the nitroblue tetrazolium reduction technique previously described by Beauchamp and Fridovich [61]. Results obtained were normalized to the protein content and expressed as units per milligram of protein.

#### Determination of Reduced Glutathione Level

Levels of intracellular glutathione (GSH) were assessed spectrofluorimetrically by monochlorobimane staining [62] and expressed as arbitrary units (AU) of fluorescence intensity.

#### Determination of Glutathione Peroxidase and Glutathione Reductase Activity

Glutathione peroxidase (GPx) and GRx activities were quantified using Glutathione Peroxidase Assay Kit (Cayman 703102) and Reductase Assay Kit (Cayman 703202), respectively. The results obtained were normalized to the protein content and expressed as units per milligram of protein.

### Quantitative RT-PCR

Total RNA from cultured cells was obtained using Trizol reagent, and 1 μg was reverse transcribed with random primers (Biolabs, Ipswich, USA) and Reverse Transcriptase MLV-RT (Fisher Scientific, Courtaboeuf, France), according to the manufacturers’ instructions. Quantitative PCR was performed with standard protocols using SYBRGreen (ABgene, Portsmouth, USA) as the fluorescent detection dye in an ABI PRISM 7000, which also contained 300 nM of primers (except for PLP and MAG with 100 nM of primers) and 50 ng of reverse transcribed RNA in 384-well plates. Melting curve analysis was applied to characterize the generated amplicons. Each reaction was performed in triplicate, and the mean of at least three independent experiments was calculated. Results were normalized to 26S mRNA levels and calculated using the ΔΔCt method. The results are expressed as 2^−ΔΔCt^ [ΔΔCt = ΔCt gene of interest − ΔCt reference gene]. The primer sequences used in real-time PCR are listed in the Supplemental Table 1*.*

### Western Blot Assay

Proteins were extracted and separated as previously described [54]. The following concentrations of primary antibodies were used: anti-PLP (1:1000), anti-MBP (1:500), anti-ERK1/2 (1:500), anti-P-ERK1/2 (1:500), anti-JNK (1:500), anti-P-JNK (1:500), anti-p38 (1:500), anti-P-p38 (1:500), and anti-β-actin (1:10000); followed by appropriate horseradish peroxidase coupled secondary antibody (anti-rabbit: 1:20,000). Specific bands were detected with the Pierce®ECL 2 Western Blotting substrate kit, using ImageQuant LAS4000 (GE Healthcare Life Sciences, Chicago, USA). Relative protein amounts were quantified with ImageJ 1.48v software and normalized to β-actin.

### Immunolabeling

At time 0 h after stretching, samples were fixed with 4% paraformaldehyde (PFA) at RT for 40 min for organotypic slices and 25 min for cells. After fixation, membranes were cut from the Bioflex® plates.

Organotypic slices were incubated for 1 h in 0.1 M L-lysine diluted in PBS-GTA (PBS with 0.2% gelatin, 0.25% Triton-X, 0.1% sodium azide) blocking solution and then incubated with primary antibodies overnight at 4 °C. The following concentrations of primary antibodies were used: anti-MAG (1:1000), anti-APP (1:700), anti-SMI-312 (1:1000), anti-Caspr (1:1000). Samples were then incubated with secondary antibodies for 2 h on a rocker at RT at the following concentrations: Alexa 488 goat anti-mouse IgG (1:1000) and/or Cy3 goat anti-rabbit (1:500), both diluted in PBS-GTA.

Cells were permeabilized with 0.1% Triton-X 100-PBS for 15 min and then incubated with primary antibodies overnight at 4 °C in PBS-BSA 5%. The following concentrations of primary antibodies were used: anti-MAG (1:250), anti-PDGFR-α (1:500), anti α-tubulin (1:4000). Samples were then incubated with secondary antibodies for 1 h on a rocker at RT at the following concentrations: Alexa 488 goat anti-mouse IgG (1:200) and/or Cy3 goat anti-rabbit (1:100), both diluted in PBS-GTA. TRITC-phalloidin (1:2000) and Hoechst (1:50,000) were used to label F-actin and cell nuclei, respectively.

All samples were mounted with Fluoromount (Southern Biotech, Birmingham, USA).

### Immunofluorescence Analysis

All images were acquired using a Zeiss confocal microscope LSM710 for cells or LSM510 for organotypic cerebellar slice and then processed with LSM Image Browser (version 4.2).

For the organotypic cerebellar slice cultures, the analysis of APP accumulation was performed on slices co-immunostained with APP and SMI-312. The number of APP aggregates was quantified per slice, for a total of 4–8 slices per condition from three independent experiments. The analysis of paranodal junctions was performed on slices co-immunostained with Caspr and MAG on images acquired for a total of 4–6 slices per condition from four independent experiments, as previously reported [63, 64].

For the oligodendrocyte-enriched primary culture, the number of Hoechst^+^ cells, the percentage of MAG^+^ and PDGFR-α^+^ cells, and also the area of MAG^+^ and PDGFR-α^+^ cells were determined using ImageJ 1.48v software. All analyses were performed in 10–30 different fields (0.26 nm^2^), randomly selected while avoiding the edge of the membrane, per condition, and from at least three independent experiments.

### Statistical Analysis

Unpaired *t* tests were calculated for two-data set when data followed a normal distribution. Otherwise, the Mann-Whitney test was performed, which does not require the assumption of normal distributions. To test for normality, the d’Agostino-Pearson test was used. All statistical analyses were performed with GraphPad Prism6 (San Diego, USA). The criterion for statistical significance was *p* < 0.05 (*: *p* < 0.05; **: *p* < 0.01; ***: *p* < 0.001). Data are expressed as mean values of *n* experiments ± SEM.

## Results

### Effects of Mechanical Tensile Strain of High Magnitude on Organotypic Culture of Cerebellar Slices

#### Mechanical Strain-Induced Axonal Injury and an Elongation of Paranodal Junctions Ex Vivo

**Fig. 1:**
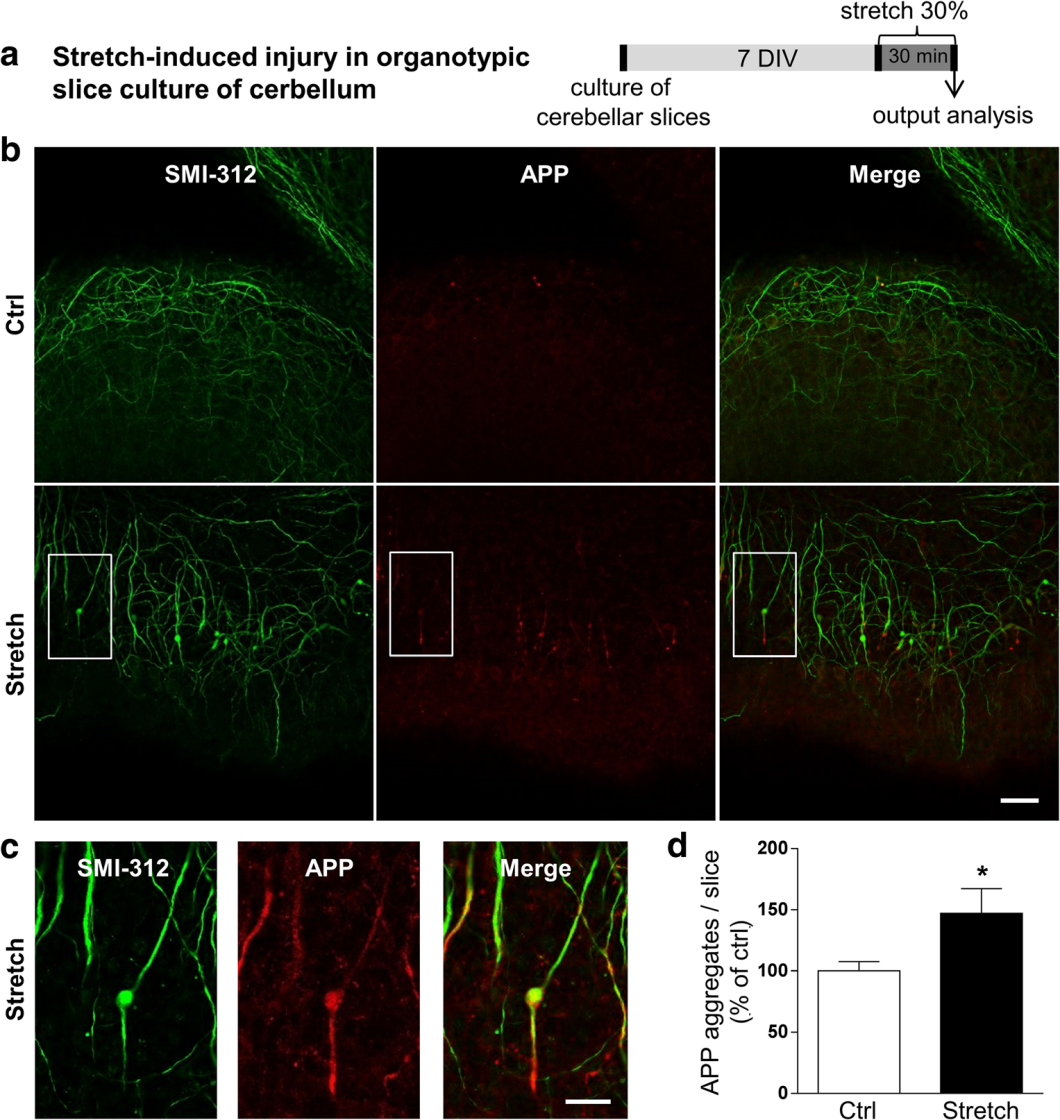
Mechanical tensile strain of 30% induces axonal injury in organotypic cerebellar slice culture. **a** Ex vivo culture and 30% strain model using organotypic slice cultures of cerebellum. **b** Double immunostaining of organotypic cerebellar slices for APP in red and SMI-312 (pan-axonal neurofilament marker) in green. Images represent control (Ctrl) and stretched (Stretch) cultures. The response of slices was evaluated at time 0 h post-stretch. Scale bar, 50 μm. **c** High magnification of the white square in the stretch panel from **b**. Scale bar, 20 μm. **d** Quantitative analysis of the number of APP aggregates per slice. Results represent the mean ± SEM (*n* = 3). *p < 0.05. APP amyloid precursor protein, DIV days in vitro

In our study, we aimed to mimic the effect of a sustained tensile strain. We chose half-an-hour static tensile strain of 30% (Fig. 1a) to induce two hallmarks of brain trauma, specifically axonal injury and elongation of paranodal junctions in a myelinated tissue.

First, we assessed the axonal injury by co-immunolabeling of cerebellar slices with APP and SMI-312 antibodies (Fig. 1b). The number of axonal APP aggregates was significantly increased in stretched slices (+ 47%, *p* < 0.05) (Fig. 1c, d), and the co-immunolabeling of SMI-312, a marker of axonal neurofilament, indicated an axonal swelling (Fig. 1b, c) in stretched slices.

**Fig. 2:**
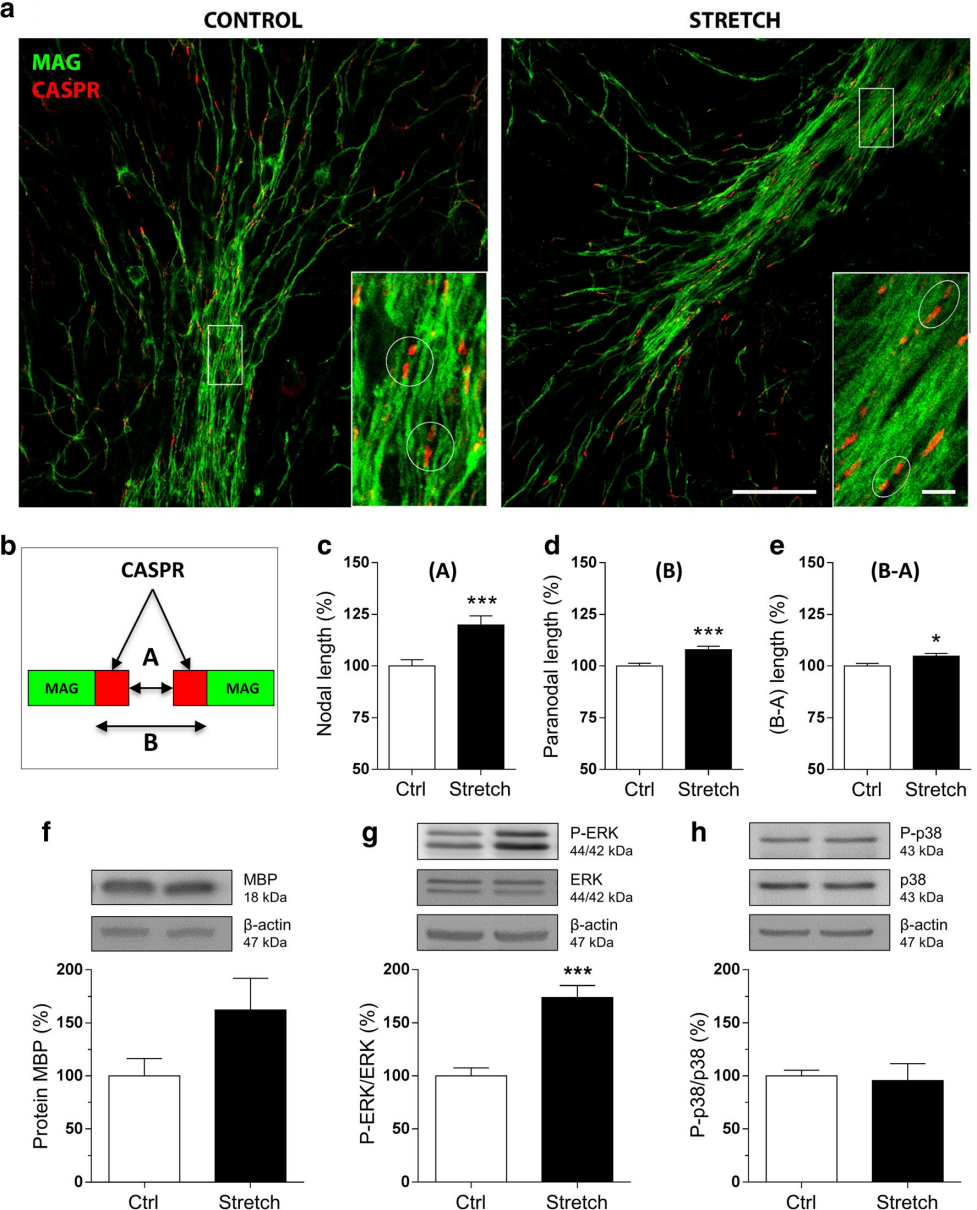
Mechanical tensile strain of 30% induces an elongation of paranodal junction and alteration in MAPK signaling in organotypic cerebellar slice culture. **a** Double immunostaining of organotypic cerebellar slices for CASPR in red and MAG in green. The response was evaluated at time 0 h post-stretch. Altered paranodal junctions are highlighted within white circles in high-magnification images. Scale bar, 50 μm; inset scale bar, 5 μm. **b** Sketch of CASPR and MAG labeling in the paranodal junction. Quantitative analysis is characterized by three length measurements: **c** nodal length called (A); **d** paranodal length called (B); and **e** (B-A) corresponding to the total length of the CASPR labeling. **f**–**h** Proteins were extracted following strain of 30% from control (Ctrl) and stretched (Stretch) slices and western blot was performed for the myelin protein MBP (**f**), ERK1/2 (**g**), and p38 (**h**). The ratio P-ERK on total ERK and the ratio P-p38 on total p38 are presented. β-Actin was used as a loading control. Results represent the mean ± SEM (*n* = 3). ***p < 0.001. CASPR contactin-associated protein, MAG myelin-associated glycoprotein

Second, we investigated the structure of the paranodal junctions by labeling the axonal membrane protein Caspr, an integral component of the paranodal complex, and the myelin sheaths by MAG, a myelin protein. Paranodal junctions are located between the nodes of Ranvier and the juxtaparanodal junctions. The paranodal complex is a structure that connects myelin and the axonal membrane at this region. The co-immunolabeling of MAG allowed us to better visualize the myelinated fibers with their pair of immunolabeled Caspr delimiting each node of Ranvier. Our results showed a significant alteration in Caspr staining in stretched slices compared to control slices (Fig. 2a). In particular, we quantified the nodal (A) length (+ 19.8%, *p* < 0.001), the paranodal (B) length (+ 7.9%, *p* < 0.001), and the (B-A) length (+ 4.7%, *p* < 0.05) (Fig. 2b–e), showing an increase of all these parameters in stretched slices. Our findings suggest an alteration of the paranodal junctions that start immediately after stretch.

#### Effect of Mechanical Strain on Myelin Protein Expression and MAPK Signaling Pathway Ex Vivo

To evaluate the signaling alterations after mechanical stretch, we performed another set of experiments and extracted proteins from cerebellar slices. First, we investigated the expression of one of the major myelin protein, MBP by western blot. The amount of MBP was not significantly altered immediately after stretch even if a tendency of increase was observed (Fig. 2f).

Second, we studied the activation of MAPK signaling pathway which includes three principal subgroups of proteins, extracellular signal-regulated kinases (ERKs), c-Jun NH2-terminal kinases (JNKs), and p38 [65–68], which are known to be responsive to stretch-induced injury and to be involved in myelin protein expression. We focused particularly on the effect of stretch on p38 (43 kDa) and ERK1/2 (44 and 42 kDa) in cerebellar slices (Fig. 2g, h). Our results showed that stretch had no effect on P-p38/p38 (Fig. 2h) but it increased the ratio of P-ERK/ERK by 74.1% (*p* < 0.001) (Fig. 2g). These results suggest an activation of MAPK-ERK following mechanical stretch injury in cerebellar slices.

### Morphological and Molecular Alterations After Mechanical Tensile Strain of High Magnitude on Isolated Oligodendrocytes

#### Mechanical Strain Provoked Marked Alterations of Oligodendrocyte Morphology In Vitro

**Fig. 3:**
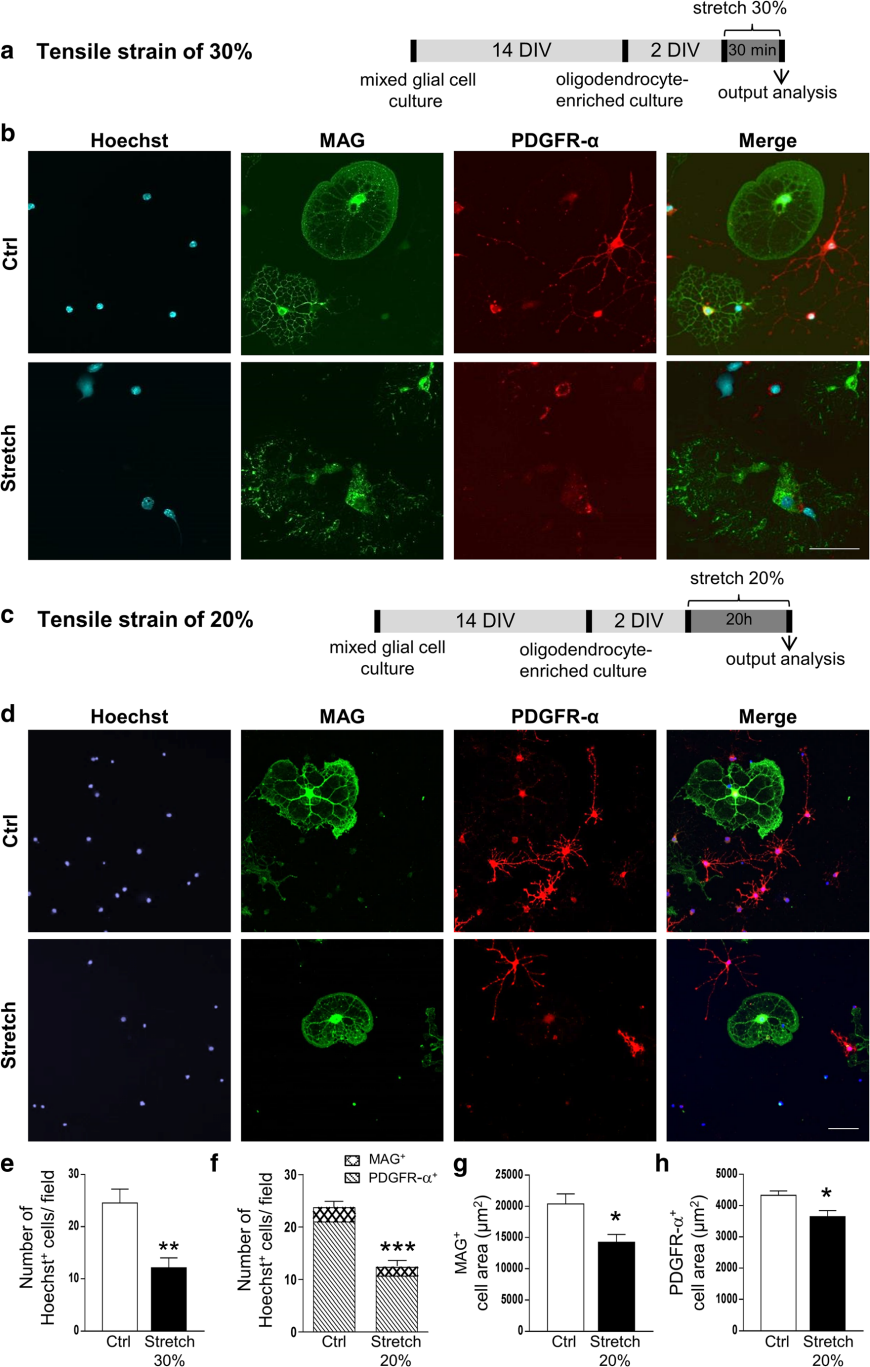
Oligodendrocyte morphology and cell adhesion after in vitro mechanical tensile strain of 20% and 30%. **a**, **c** In vitro culture and strain model protocol of oligodendrocyte-enriched primary culture for mechanical strain of 30% and 20%, respectively*.* **b**, **d** Immunostaining of mature oligodendrocytes (MAG in green), their precursors (PDGFR-α in red), and nuclei (Hoechst in blue) in control (Ctrl) and stretched (Stretch) cells after mechanical strain of 30% and 20%, respectively. Scale bar, 100 μm. **e** Quantitative analysis of the number of Hoechst^+^ cells per field (0.26 nm^2^) after 30% of strain. **f** Quantitative analysis of the number of Hoechst^+^ cells per field (0.26 nm^2^) and of the percentage of MAG^+^ and PDGFR-α^+^ cells after 20% of strain. **g**, **h** Quantitative analysis of the area of MAG^+^ cells (**g**) and PDGFR-α^+^ cells (**h**) after 20% of strain. Results represent the mean ± SEM (*n* = 3). *p < 0.05; **p < 0.01; ***p < 0.001. DIV days in vitro

We assessed the effect of 30% strain for 30 min on the morphology of oligodendrocyte using oligodendrocyte-enriched primary culture. All analyses were performed immediately after the end of stretching period (Fig. 3; Fig. S1).

Double immunostaining either with markers of cytoskeleton (actin and α-tubulin) or oligodendrocytes (MAG, PDGFR-α) revealed profound cellular damage with loss of oligodendrocyte ramifications and also partial cell loss after mechanical strain of 30% for 30 min (Fig. S1a; Fig. 3a, b, e). As the damage was detrimental to the isolated oligodendrocytes in primary culture, we also used a lower magnitude of strain (20% for 20 h; Fig. 3c), after which cells appeared retracted (Fig. S1b; Fig. 3d).

Applied strain of both magnitudes induced a decrease in the number of Hoechst^+^ adherent cells by 50% (*p* < 0.001) (Fig. 3e, f), which lasted at least 24 h after stress induction (not shown). In our experimental conditions of primary culture, we obtained more immature than mature oligodendrocytes (80–90% immature PDGFR-α^+^ and 10–20% mature MAG^+^), as expected [69]. Interestingly, the lowest strain magnitude (20%) did not alter the percentage of mature MAG^+^ (11.50 ± 0.29 versus 13.50 ± 0.87% cells/field) and immature PDGFR-α^+^ (86.33 ± 0.88 versus 80.33 ± 3.28% cells/field) oligodendrocytes (Fig. 3f), suggesting that there were less Hoechst^+^ adherent cells with the same proportion of mature and immature cells. In addition, the surface area of MAG^+^ or PDGFR-α^+^ cells was reduced following strain of 20% (Fig. 3g, h), in accordance with our data on cytoskeleton (Fig. S1). In the case of 30% strain, oligodendrocytes had an elongated nuclei shape with no cell ramifications (Fig. 3b). It was impossible to identify cells as mature MAG^+^ or immature PDGFR-α^+^ oligodendrocytes. Thus, we were not able to provide the analysis of cell surface and ramifications after 30% strain.

#### Mechanical Strain Altered the Expression of Myelin Proteins and MAPK Signaling Pathways In Vitro

**Fig. 4:**
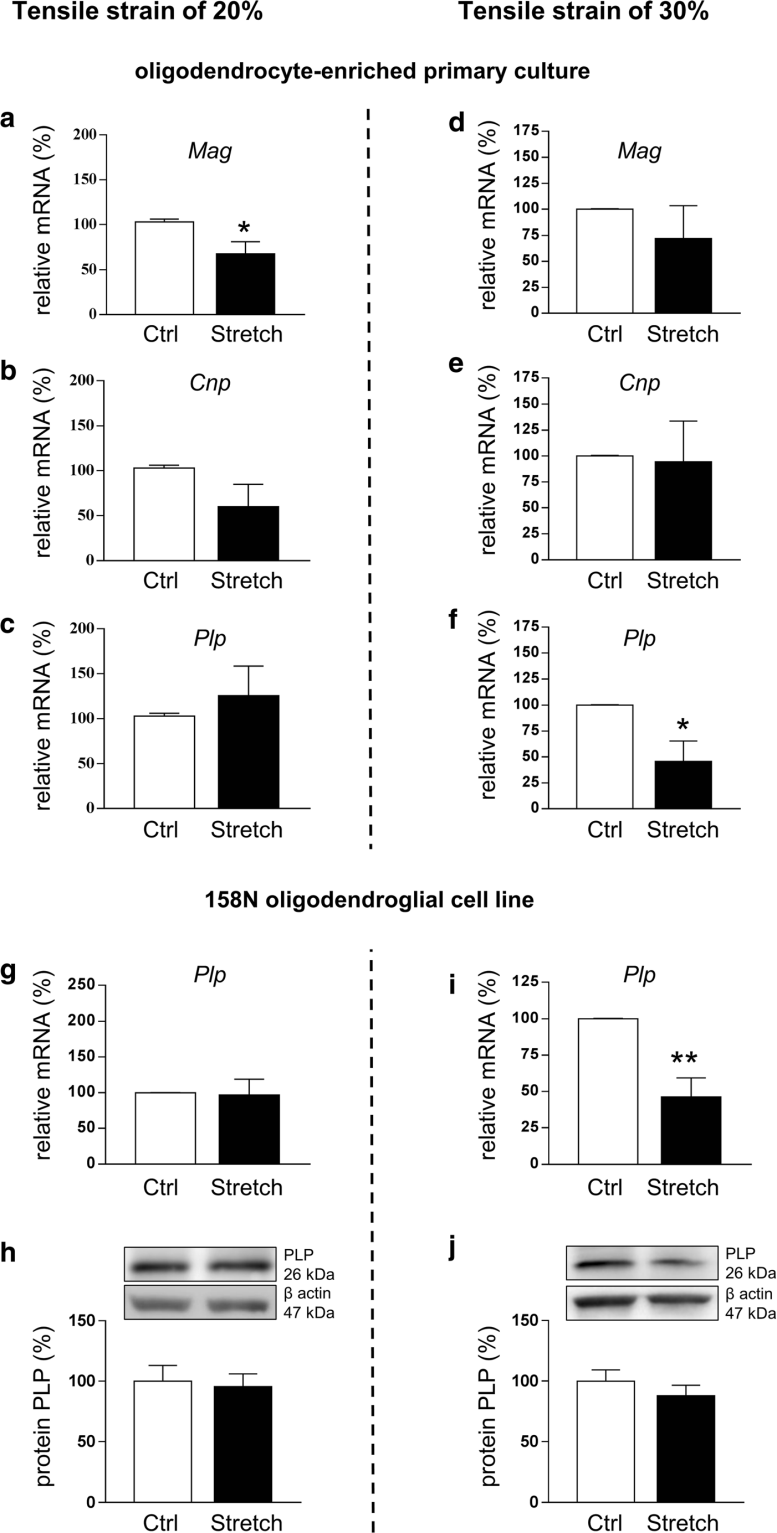
Alteration in myelin gene expression after in vitro mechanical tensile strain of 20% and 30%. Total RNA was extracted and RT-qPCR was performed in control (Ctrl) and stretched (Stretch) cells for myelin genes *Mag* (a, d), *Cnp* (b, e), and *Plp* (c, f, g, i) after strain of 20% (a, b, c, g) and 30% (d, e, f, i). Results represent the mean ± SEM of three independent experiments performed in triplicate. *p < 0.05; **p < 0.01. (h, j) Proteins were extracted from control (Ctrl) and stretched (Stretch) 158N cells and western blot was performed for myelin protein PLP (26 kDa) following 20% (h) and 30% (j) of strain. β-Actin was used as a loading control. Results represent the mean ± SEM of three independent experiments

The effect of stretch was also assessed at the level of myelin proteins. The mRNA level of *Mag* decreased by 30% (*p* < 0.05) (Fig. 4(a)); however, no significant changes for *Cnp* (Fig. 4(b)) and *Plp* (Fig. 4(c)) were observed following 20% strain. In contrast, *Plp* expression was decreased by 55% (*p* < 0.05) (Fig. 4(f)), with no significant changes for *Mag* and *Cnp* (Fig. 4(d, e)) following 30% strain.

In the 158N oligodendroglial cell line, strain of 20% did not affect PLP at the mRNA (Fig. 4(g)) and protein levels (Fig. 4(h)), while strain of 30% significantly decreased PLP by 60% (*p* < 0.01) at the mRNA level (Fig. 4(i)) without an effect on protein level (Fig. 4(j)). Hence, strain of both magnitudes altered the expression of some myelin protein genes, and interestingly, we observed the same impact of strain magnitude on *Plp* expression in oligodendrocytes either in primary cell culture or in the 158N cell line.

**Fig. 5:**
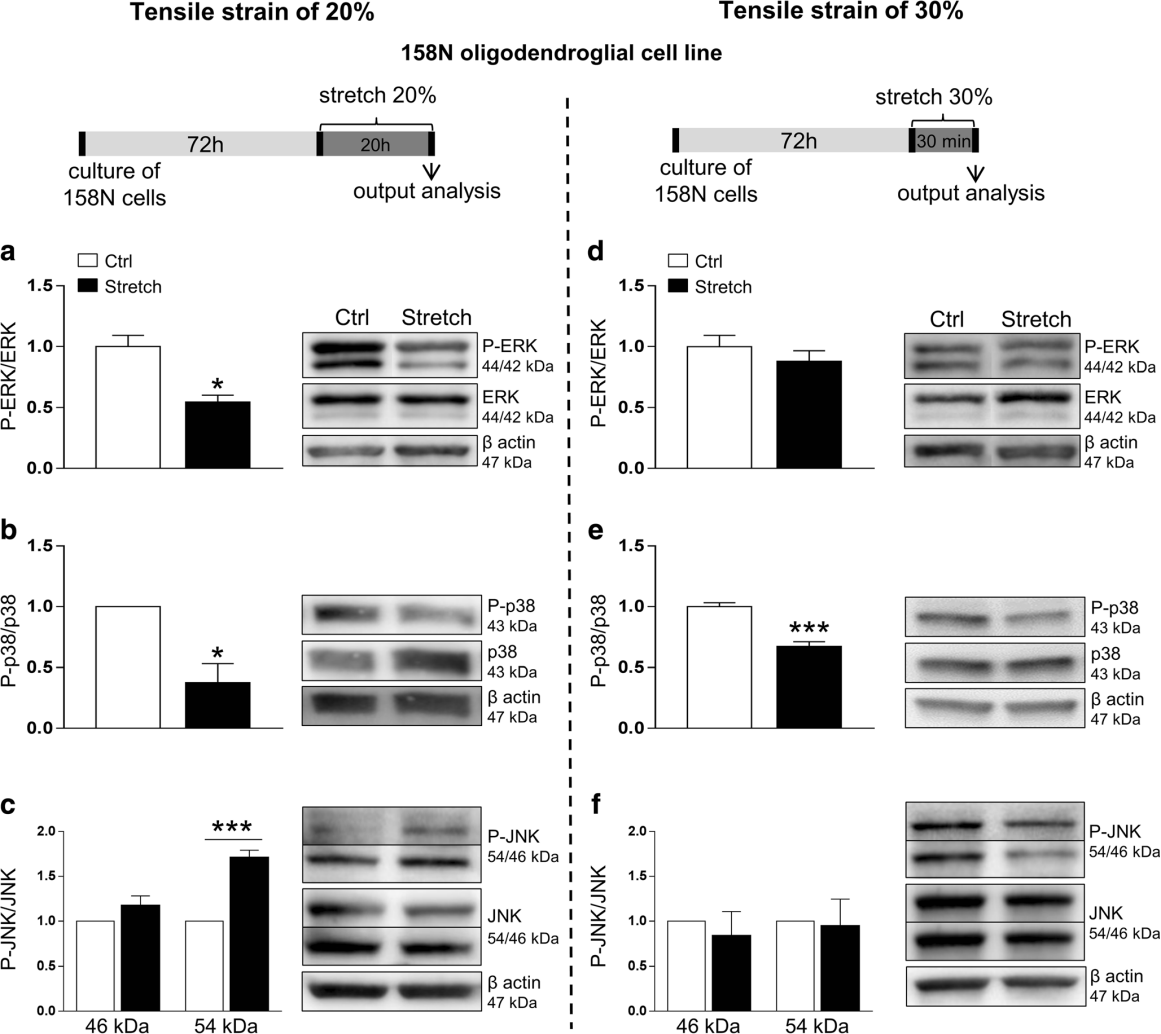
Alteration in MAP kinases ERK1/2, p38, and JNKs signaling after tensile strain of 20% and 30% in the 158N cell line. Proteins were extracted from control (Ctrl) and stretched (Stretch) cells and western blot was performed for ERK1/2 (a, d), p38 (b, e), and JNKs (c, f). β-Actin (47 kDa) was used as a loading control. The ratio of P-ERK to total ERK and the ratio of P-p38 to total p38 are presented following 20% (a, b, respectively) and 30% (d, e, respectively) of strain. The ratio of P-JNK to total JNK is presented following strain of 20% (c) and 30% (f) for the 54 and 46 kDa isoforms. Results represent the mean ± SEM of three independent experiments. *p < 0.05; ***p < 0.001

We also attempted to study the MAPK signaling pathways in the 158N cell line. Mechanical strain of 20% reduced the ratio of P-ERK/ERK by 50% (*p* < 0.05) while the strain of 30% did not have an effect on ERK signaling (Fig. 5(a, d)). Both 20% and 30% strains decreased the ratio of P-p38/p38 by 60% (*p* < 0.05) and by 30% (*p* < 0.001), respectively (Fig. 5(b, e)). Finally, 20% strain increased the ratio of P-JNK/JNK for the 54 kDa isoform (+ 70%, *p* < 0.001) (Fig. 5(c)), while 30% strain did not affect this ratio (Fig. 5(f)).

#### Mechanical Strain Provoked Increased Level of Intracellular ROS and Oxidized Proteins and Altered the Anti-oxidant Systems In Vitro

**Fig. 6:**
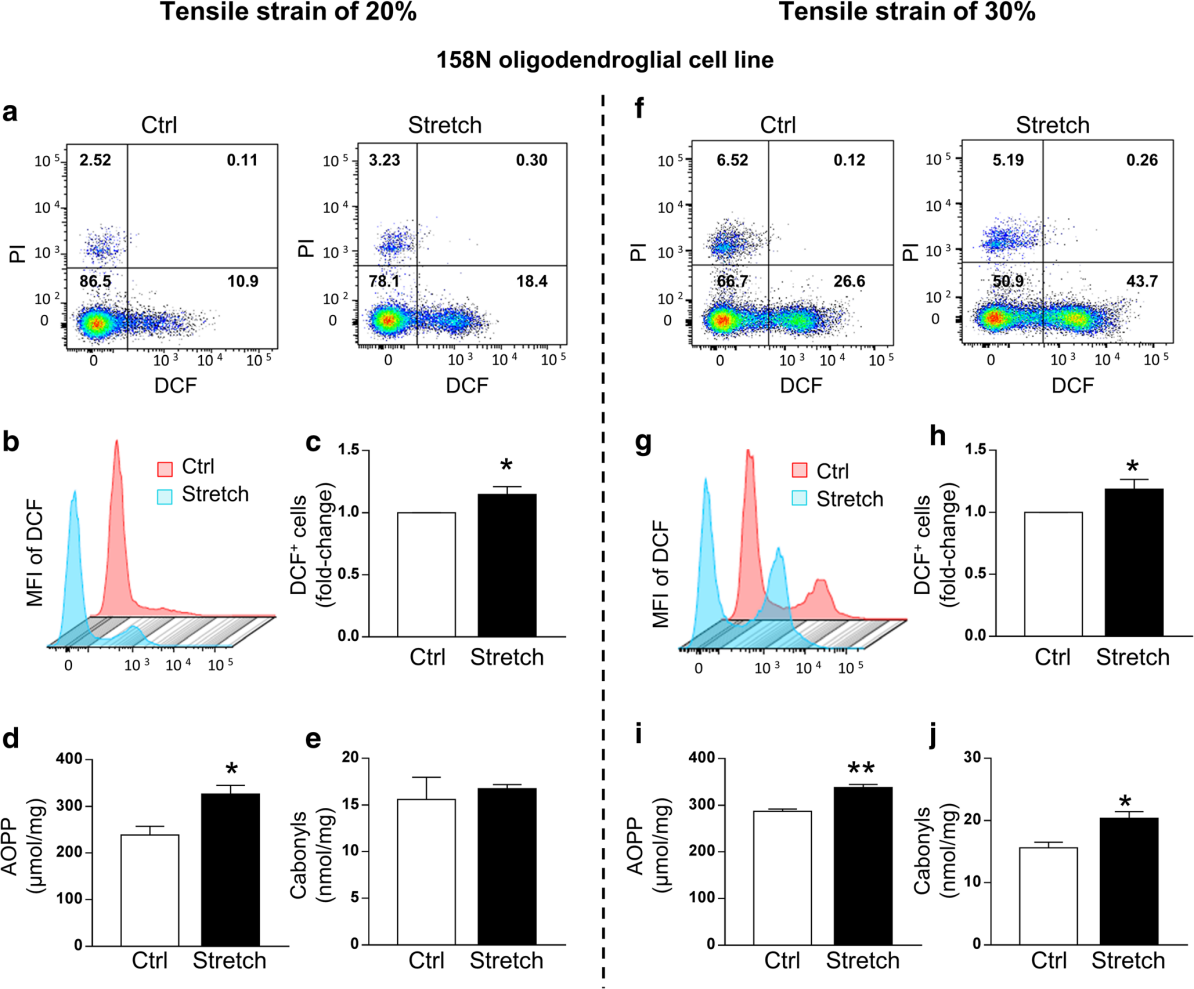
Stretch increases the level of intracellular ROS in the 158N cell line after tensile strain of 20% and 30%. The intracellular ROS level was detected by the DCFH-DA fluorescent probe. Representative DCF-stained (FITC channel) and PI-stained (PE channel) pictures of control (Ctrl) and stretched (Stretch) cells following strain of 20% (a) and 30% (f). Representative picture of the mean fluorescence intensity (MFI) of DCF measured in DCF^+^/PI^−^ cells following strain of 20% (b) and 30% (g). Quantitative analysis of the percentage of DCF^+^ cells following strain of 20% (c) and 30% (h). Results are expressed as the fold change in comparison to the control group. Values are the mean ± SEM of three experiments performed in triplicate. AOPP (μmol/L) and carbonyls (nmol/mg) were measured in control (Ctrl) and stretched (Stretch) cells following 20% (d, e) and 30% (i, j) of strain. Results represent the mean ± SEM (*n* = 5). *p < 0.05; **p < 0.01. DCF 2′,7′ fluorescent dichlorofluorescein. PI propidium iodide

**Fig. 7:**
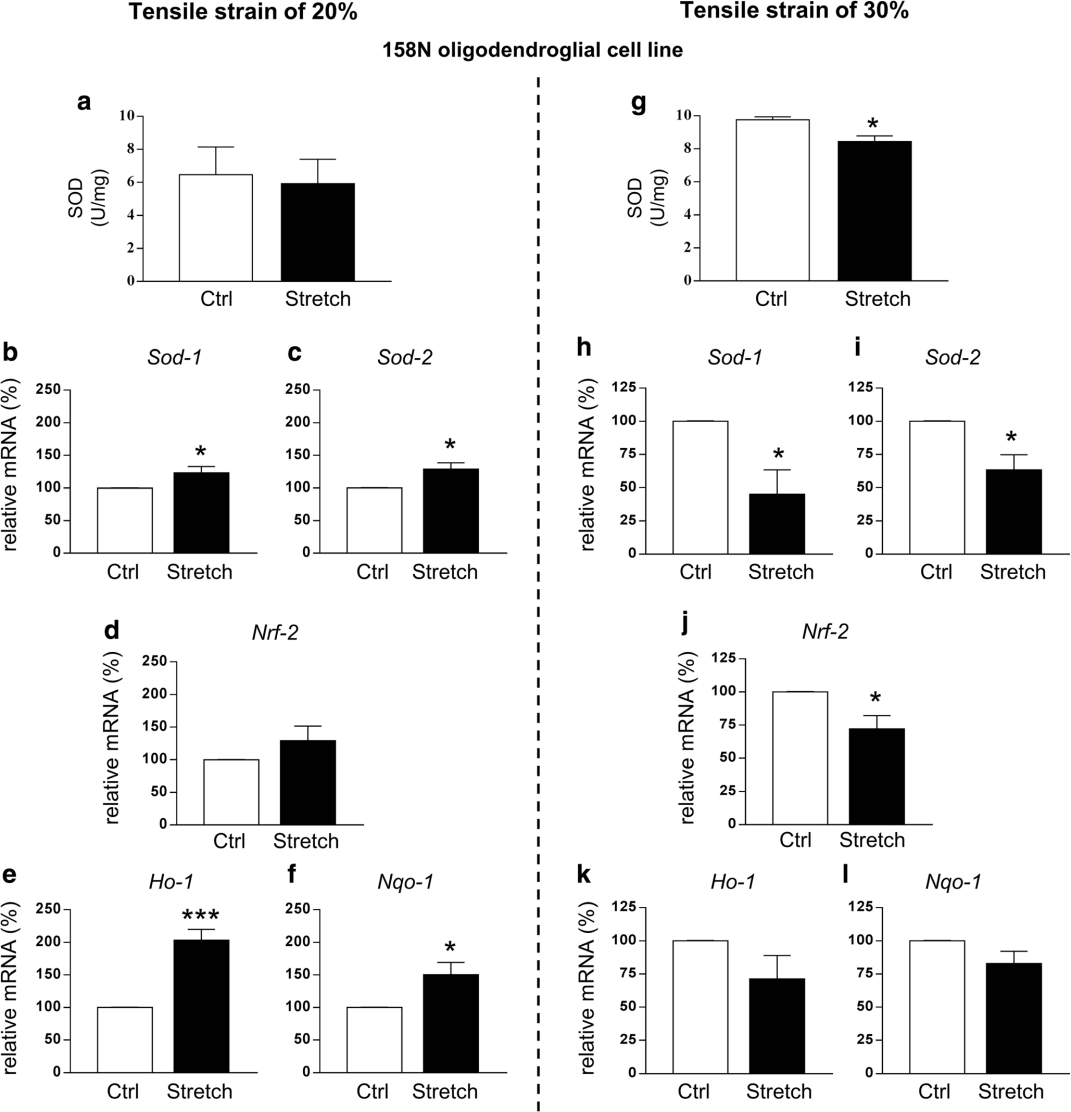
Effect of stretch on different oxidative stress parameters in the 158N cell line after tensile strain of 20% and 30%. SOD activity was measured in control (Ctrl) and stretched (Stretch) cells following strain of 20% (a) and 30% (g). Results represent the mean ± SEM (*n* = 5). Total RNA was extracted and RT-qPCR was performed in control (Ctrl) and stretched (Stretch) cells following 20% and 30% of strain for antioxidant genes *Sod-1* (b, h), *Sod-2* (c, i), *Nrf-2* (d, j), *Ho-1* (e, k), and *Nqo-1* (f, l). Results represent the mean ± SEM of three independent experiments performed in triplicate. *p < 0.05; ***p < 0.001

The morphological alterations and changes in myelin proteins observed in oligodendrocytes prompted us to further investigate the molecular mechanisms related to oxidative stress induced by mechanical strain. To achieve this objective, we chose the 158N oligodendroglial cell line, as the appropriate cellular model, to perform biochemical assays and FACS analysis. 158N cells were exposed to mechanical strain of 20% and 30%, and all analyses were performed immediately after the end of the stretching period (Figs. 6 and 7).

We used FACS to assess the effect of mechanical strain of high magnitude on cell death and ROS production. In control and stretched cells, the number of PI^+^ cells was less than 7% regardless of group condition and of stretch severity showing no effect of stretch on cell death in 158N cells (Fig. 6(a, f)). ROS production, evaluated by assessing the redox-sensitive intracellular conversion of H2DCFDA to DCF, was significantly increased in stretched cells compared to control. Stretch induced an increase in the number of DCF^+^/PI^−^ cells either after strain of 20% (+ 15%, *p* < 0.05) or 30% (+ 20%, *p* < 0.05) (Fig. 6(b, c, g, h)). We next investigated whether the amount of ROS produced during the stretch period alterated cellular constituents, such as oxidation of proteins, evaluated by the amount of advanced oxidation protein products (AOPP) and carbonyls. The amount of AOPP in stretched cells was significantly increased compared to the control group after strain of 20% (326 ± 19.05 versus 239 ± 18.62 μmol/mg; *p* < 0.05) and 30% (338 ± 6.57 versus 287 ± 5.04 μmol/mg; *p* < 0.01) (Fig. 6(d, i)). The amount of carbonyl groups did not change after 20% strain (Fig. 6(e)), but it increased after 30% strain (20.40 ± 1.06 versus 15.63 ± 0.90 nmol/mg; *p* < 0.05) (Fig. 6(j)).

We further investigated the effect of strain on anti-oxidant systems in 158N cell line. Strain of 20% provoked a decrease in the level of reduced GSH, one of the major antioxidant molecules, accompanied by a decrease in activity of glutathione peroxidase (GPx), the enzyme that breaks down H_2_O_2_ into H_2_O (Table 1). After moderate injury, the level of GSH was markedly decreased accompanied by a reduction of GPx activity. Level of oxidized glutathione (GSSG) was also assessed, but it was not detectable in our system analysis. Glutathione reductase (GR) was not affected regardless of group condition and stretch severity (Table 1). We decided to further analyze the status of the antioxidant systems in our cells (Fig. 7). The activity of superoxide dismutase (SOD), a major antioxidant enzyme, did not change in stretched cells after 20% strain (Fig. 7(a)), while it decreased after 30% strain (9.78 ± 0.17 U/mg for control versus 8.43 ± 0.35 U/mg for stretch condition, *p* < 0.05) (Fig. 7(g)). The effect of stretch was also investigated at the mRNA level of the cytoplasmic form of this enzyme, *Sod-1*, and the mitochondrial form, *Sod-2*. Stretch induced an increase of both *Sod-1* and *Sod-2* expression after 20% strain (Fig. 7(b, c)), while an opposite response was observed after 30% strain with a decrease in the expression of both *Sod-1* and *Sod-2* (Fig. 7(h, i)).

**Table 1:** Effect of stretch on glutathione and enzymes involved in glutathione redox cycle in the 158N cell line after tensile strain of 20% and 30%

	Ctrl	Stretch	*p* value
20% strain model			
GSH (AU)	403.7 ± 9.02	363.3 ± 4.41*	0.0159
GPx (U/mg)	0.392 ± 0.039	0.216 ± 0.024**	0.0020
GR (U/mg)	0.018 ± 0.005	0.050 ± 0.020	0.1408
30% strain model			
GSH (AU)	421.2 ± 10.13	370.8 ± 7.51**	0.0026
GPx (U/mg)	0.327 ± 0.014	0.272 ± 0.015*	0.0247
GR (U/mg)	0.013 ± 0.003	0.030 ± 0.006	0.0668

Reduced (GSH) and oxidized (GSSG) glutathione, glutathione peroxidase (GPx), and glutathione reductase (GR) were measured in control (Ctrl) and stretched (Stretch) cells following strain of 20% and 30%. GSSG was not detectable in our system. Results represent the mean ± SEM (*n* = 9 for 20% and *n* = 6 for 30%)

*p < 0.05; **p < 0.01

Next, we evaluated the expression of Nrf2, a transcription factor that regulates the expression of many phase II detoxifying and antioxidant enzymes, such as heme oxygenase 1 (HO-1) and NAD(P)H:quinone oxidoreductase 1 (NQO-1) [70]. Strain of 20% did not affect *Nrf2* expression at the mRNA level (Fig. 7(d)) while strain of 30% decreased levels of the *Nrf2* transcript (Fig. 7(j)). We also observed a significant increase of *Ho-1* (+ 100%, *p* < 0.01) and *Nqo-1* (+ 50%, *p* < 0.05) mRNA levels after 20% strain (Fig. 7(e, f)), while no effect was measured after 30% strain (Fig. 7(k, l)).

## Discussion

The aim of this work was to decipher the molecular responses of oligodendrocytes and myelinated fibers, and the fate of myelin proteins following a traumatic event induced by a mechanical strain of high magnitude mimicking brain trauma. We developed several models of stretch-induced injury, ex vivo in organotypic cerebellar slice cultures and in vitro in isolated oligodendrocytes (primary culture, and 158N cell line).

Stretched cerebellar slices showed an accumulation of amyloid precursor protein (APP), a marker of axonal injury, and axonal swelling, a sign of altered axonal transport [71]. APP accumulation within axons is recognized as the gold standard for the identification of axonal injury [42, 43]. Therefore, the mechanical strain of 30% applied to our culture of cerebellar slices was sufficient to induce an axonal injury that is a hallmark of brain trauma. In addition, the structure of the paranodal junctions, that flank the nodes, appeared more “elongated” in stretched slices than in control slices suggesting an alteration of the paranodal junctions immediately after stretch. This observation is in line with a study in which paranodal myelin damage was reported in an ex vivo model of stretch-induced injury in guinea pig spinal cord [45]. Another study showed that primary paranodal demyelination modulates axonal depolarization in a model of axonal injury [72]. The paranode regions contain the voltage-gated potassium channels in high density and the injury-induced paranodal demyelination modulates potassium accumulation and membrane depolarization that, in turn, affect the axonal transition to degradation. The authors proposed that the extent of paranodal demyelination should be considered as an “injury parameter” that is likely to determine the stability of axonal function in the temporal window of minutes post trauma [72]. Moreover, the width of the nodes of Ranvier is crucial for a fast conduction. In fact, in normal myelinated axons, the conduction velocity is inversely related to the node width. Taken together, our results showed that stretch induced an alteration of paranodal junctions immediately after the stretch that could initiate demyelination.

Among the in vitro models of traumatic injury, several reports addressed the effect of stretch-induced injury on different CNS cell types including neurons, astrocytes, and microglia. It has been demonstrated that such injury results in structural changes in both glia and neurons [29, 73–75]. Generally, the extent of damage is proportional to the degree of strain [76, 77]. Recent finding showed that stretch-induced mechano-stimulation of oligodendrocyte progenitor cells, in a physiological range of tensile strain (10–15%), is able to promote cell differentiation. However, the effect of tensile strain on oligodendrocytes at higher magnitude (20–30%), which occurs following brain injury [40, 41], remains unknown. Therefore, our aims were to study the effect of such injury on oligodendrocyte morphology and cell loss, and to decipher the subsequent cellular and molecular responses to a mechanical strain of high magnitude.

We addressed the effect of mechanical strain of different magnitudes (20% or 30%) on oligodendrocyte morphology and cell loss in oligodendrocyte-enriched primary cultures. As expected, immature oligodendrocytes expressed PDGFR-α, and mature oligodendrocytes, that form “myelin-like” membranes in vitro, expressed myelin proteins such as MAG [1, 78, 79]. Our data showed a significant loss of oligodendrocytes following both strains of 20% and 30%. The lower strain of 20% did not affect the ratio of mature to immature oligodendrocytes, suggesting that the cell maturation rate was not modified in our experimental condition. Changes in cell morphology contrary to the percentage of cell loss were proportionally related to the magnitude of mechanical strain. In fact, strain of 20% caused cell retraction, while strain of 30% provoked a profound alteration of cell ramifications with disappearance of the cytoskeleton. Cells can respond to mechanical stretch by modulating their spread area, generally as a function of strain magnitude and strain frequency, as previously reported in endothelial cells [46], and in fibroblasts [80]. At the molecular level, the expression of myelin proteins was also affected following stretch with a significant decrease in the mRNA level of *Mag* (20% strain) and *Plp* (30% strain). Overall, our findings showed that stretch was able to induce an alteration of cell morphology and myelin protein expression in oligodendrocytes that was strain magnitude-dependent.

Since oligodendrocytes are particularly mechanosensitive and vulnerable to mechanical strain of high magnitude, and also vulnerable to oxidative stress [23, 24], we assessed the effect of mechanical strain of high magnitude on the production of ROS in 158N cells. While stretch did not induce cell death in 158N cells, probably due to our experimental condition (higher cell density compared to primary culture) or cell line resistance, it caused a significant increase in the production of ROS. In our model, elevated ROS levels led to an increase of AOPP after strain of 20% and 30%, and of protein carbonylation only after strain of 30%. In addition, mechanical strain caused a diminution of anti-oxidant defenses, as reflected by the decreased level of GSH, which was strain magnitude-dependent. It was also accompanied by a decrease of GPx, which detoxifies H_2_O_2_. GSH is the first line of cell defenses as a ROS scavenger. Hence, the ratio of GSH to GSSG is often used as an indicator of the oxidative stress status [81]. In our system, the significant decrease of GSH indicates an alteration in the glutathione equilibrium following mechanical strain of high magnitude.

In addition, analysis of the cellular oxidative redox status suggested that the activation of anti-oxidant systems was strain magnitude-dependent. The Nrf2 pathway, a major determinant of detoxifying phase II gene induction [70], once activated is able to induce the expression of *Ho-1* and *Nqo-1*, which protect cells from oxidative stress [82]. On one hand, strain of 20% increased the expression of anti-oxidant genes (*Sod-1*, *Sod-2*, *Ho-1*, and *Nqo-1*), showing that cells were able to activate their anti-oxidant defense system. On the other hand, strain of 30% has an opposite effect accompanied by a decreased SOD activity. Therefore, strain of 30% induced a significant alteration of the antioxidant system rendering cells more vulnerable and incapable of properly activating the antioxidant response.

We also attempted to study the effect of mechanical stretch on MAPK activation, a particularly stretch-responsive pathway. In CNS glial cells, like astrocytes, mechanical injury activates ERK [66] and p38 [68]. The role of the MAPK signaling is emphasized as a point of convergence for various signaling cascades regulating gene expression [83]. In fact, the MAPK pathway is involved in myelin protein expression, oligodendroglial development, and myelination [84–89]. Several studies showed that p38 MAPK inhibition prevents myelin gene expression and OPC differentiation [86, 90, 91]. This is in accordance with our in vitro findings where stretch-induced p38 MAPK inhibition was accompanied with a decreased expression of at least one myelin protein. However, we did not observe p38 MAPK inhibition in slices because what we observed in cerebellar slices is the final response of various cell subpopulations in the cerebellum. ERK1/2 has been also identified as critically important in regulating oligodendroglial development and myelination; in particular, it seems that ERK1/2 plays a key and direct role in promoting myelination (see review [85]). ERK1/2 was activated in cultured OPCs following treatment with rolipram, a phosphodiesterase 4 (PDE4) inhibitor, and seems to be involved in the remyelinating effect of this compound after demyelination ex vivo and in vivo [92]. The activation of ERK1/2 observed in our model of cerebellar slices could be a positive response to counteract the effect of mechanical stretch injury on myelin integrity.

Moreover, a link has been established between oxidative stress and MAPKs (see review [93, 94]). In fact, ROS can induce or mediate the activation of the MAPK signaling pathways. In general, increased ROS production leads to the activation of ERKs, JNKs, and p38. In our model, the activation of JNK after 20% strain in vitro might be related to increased ROS production. Our results confirm also that mechanical stretch was able to activate the JNK pathway, which is known to be responsive to environmental stress [65]. However, the activation of JNK pathway appears to be strain magnitude-dependent since it was only observed following 20% strain. Taken together, mechanical stretch of high magnitude disturbs MAPK signaling in oligodendrocytes and cerebellar slices, which can affect cell survival and the expression of myelin proteins.

In conclusion, we developed several models of stretch-induced injury in oligodendrocytes and cerebellar slices, showing for the first time that stretch-induced injury causes significant alterations of oligodendrocytes and myelin integrity. Mechanical stretch of high magnitude provokes axonal injury and elongation of paranodal junctions that could initiate demyelination. Overall, these models are relevant for studying the pathophysiological events that occur after stretch-induced injury as well as for testing the therapeutic compounds for white matter protection.

## Electronic Supplementary Material

Fig S1 (PNG 1537 kb)

High resolution image (TIF 6828 kb)

ESM 2 (DOCX 64 kb)
