# Supplementary figures and images for: Mechanical Stretch of High Magnitude Provokes Axonal Injury, Elongation of Paranodal Junctions, and Signaling Alterations in Oligodendrocytes
Source: Mol Neurobiol. 2018 Oct 8;56(6):4231–48. doi: 10.1007/s12035-018-1372-6 (PMC6505516; doi:10.1007/s12035-018-1372-6)

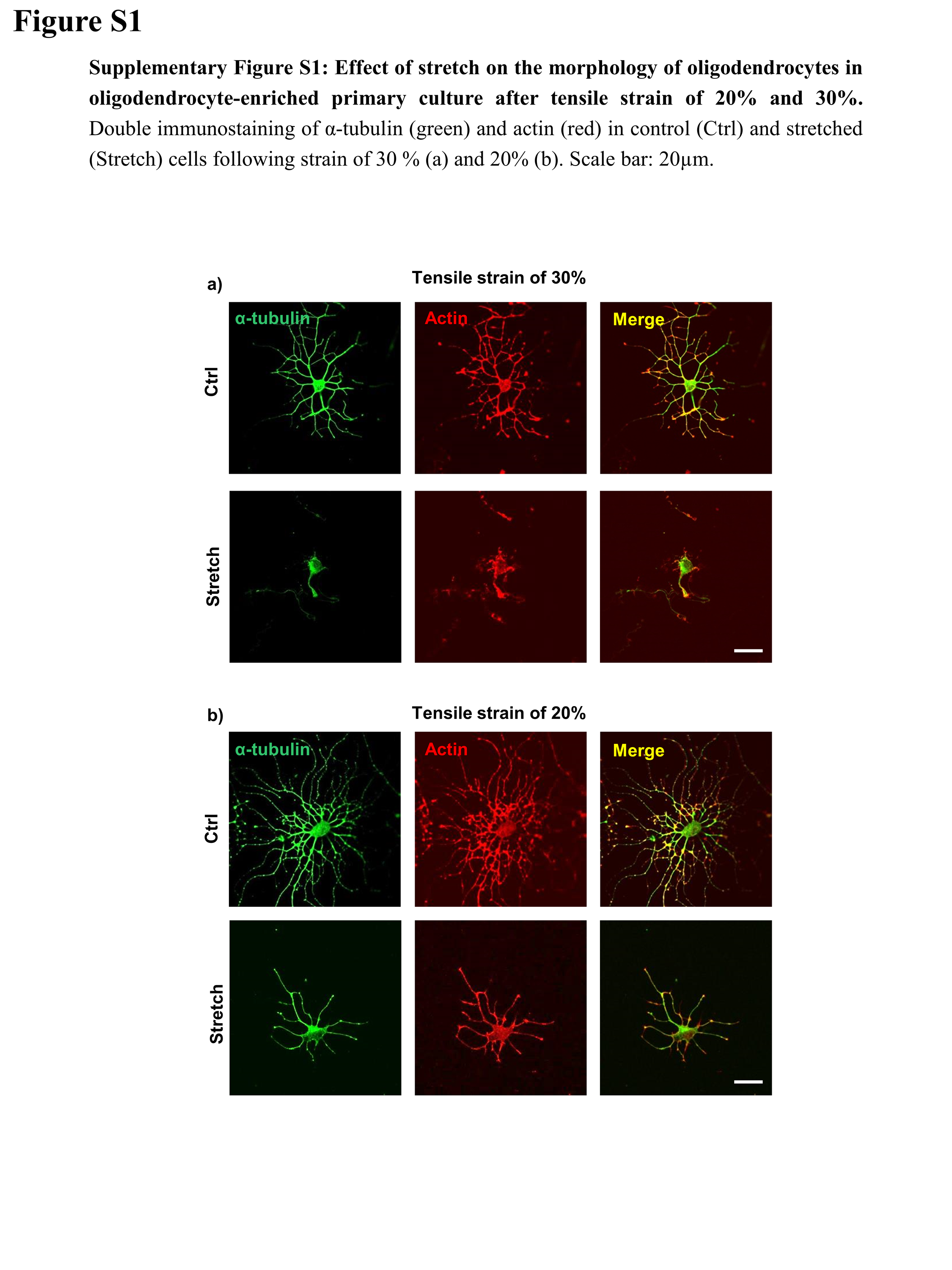

Supplement: Supplementary file 1 — (PNG 1537 kb) [file 12035_2018_1372_Fig8_ESM.png]

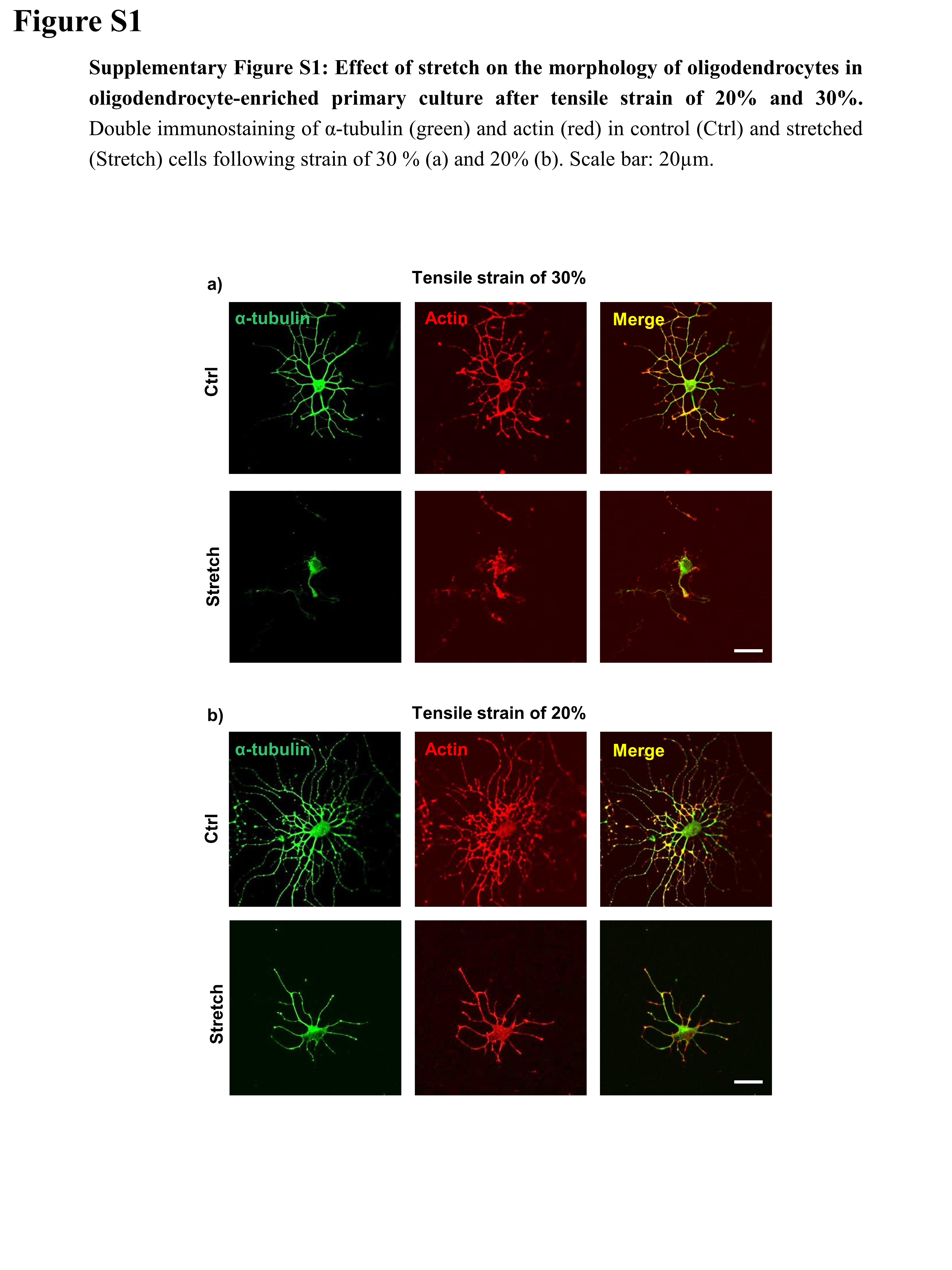

Supplement: Supplementary file 2 — High resolution image (TIF 6828 kb) [file 12035_2018_1372_MOESM1_ESM.tif]
