# Supplementary material for: Mechanical Stretch of High Magnitude Provokes Axonal Injury, Elongation of Paranodal Junctions, and Signaling Alterations in Oligodendrocytes
Source: Mol Neurobiol. 2018 Oct 8;56(6):4231–48. doi: 10.1007/s12035-018-1372-6 (PMC6505516; doi:10.1007/s12035-018-1372-6)
Supplement: Supplementary file 3 — (DOCX 64 kb) [file 12035_2018_1372_MOESM2_ESM.docx]

| ***Sod-1*** | Forward | 5’-GCC AAT GTG TCC ATT GAA GA-3’ |
| --- | --- | --- |
|  | Reverse | 5’-GTT TAC TGC GCA ATC CCA AT-3’ |
| ***Sod-2*** | Forward | 5’-GAG CTG CCT TAC GAC TAT GG-3’ |
|  | Reverse | 5’-TGA AGA GCG ACC TGA GTT G-3’ |
| ***Ho-1*** | Forward | 5’-CAC GCA TAT ACC CGC TAC CT-3’ |
|  | Reverse | 5’-CCA GAG TGT TCA TTC GAG CA-3’ |
| ***Nqo-1*** | Forward | 5’-GCG AGA AGA GCC CTG ATT GTA CTG-3’ |
|  | Reverse | 5’-TCT CAA ACC AGC CTT TCA GAA TGG-3’ |
| ***Nrf-2*** | Forward | 5’-TCT CCT CGC TGG AAA AAG AA-3’ |
|  | Reverse | 5’-AAT GTG CTG GCT GTG CTT TA-3’ |
| ***Plp*** | Forward | 5’-AGC AAA GTC AGC CGC AAA AC-3’ |
|  | Reverse | 5’-CCA GGG AAG CAA AGG GGG-3’ |
| ***Mag*** | Forward | 5'-ACT GGT GTG TGG CTG AGA AC-3' |
|  | Reverse | 5'-GGA TTA TGG GGG CAA ACT C-3' |
| ***Cnp*** | Forward | 5’- GAC AGC GTG GCG ACT AGA CT -3’ |
|  | Reverse | 5’- CAC CTG GAG GTC TCT TTC CA -3’ |
| ***26S*** | Forward | 5’- AGG AGA AAC AAC GGT CGT GCC AAA A -3’ |
|  | Reverse | 5’- GCG CAA GCA GGT CTG AAT CGT G-3’ |

**Supplemental Table 1:** Primers used for RT-qPCR analysis (obtained from Eurofins Genomics Operon, Orsay, France).
